# Dietary Supplementation of Chitosan Oligosaccharide–*Clostridium butyricum* Synbiotic Relieved Early-Weaned Stress by Improving Intestinal Health on Pigeon Squabs (*Columba livia*)

**DOI:** 10.3389/fimmu.2022.926162

**Published:** 2022-07-01

**Authors:** Jiashu Wen, Wenyan Zhao, Jiankui Li, Caihong Hu, Xiaoting Zou, Xinyang Dong

**Affiliations:** Key Laboratory of Molecular Animal Nutrition, Ministry of Education, Laboratory of Animal Feed and Nutrition of Zhejiang Province, College of Animal Sciences, Zhejiang University, Hangzhou, China

**Keywords:** chitosan oligosaccharides, *Clostridium butyricum*, synbiotic, early-weaned stress, pigeon squabs, growth performance, intestinal health

## Abstract

According to a previous study, we had found that early weaning causes harm to growth performance, intestinal morphology, activity of digestive enzymes, and antioxidant status in pigeon squabs (*Columba livia*). Chitosan oligosaccharides (COS) and *Clostridium butyricum* have been reported to have great potential to improve the growth performance and intestinal health of early-weaned animals. Therefore, the aim of this study is to explore whether dietary supplementation with COS-*C. butyricum* synbiotic could relieve early-weaned stress by evaluating its effects on growth performance and intestinal health in pigeon squabs. A total of 160 squabs (weaned at 7 days of age) were randomly divided into 5 groups: the control group, fed with artificial crop milk; the COS supplementation group, fed with artificial crop milk + 150 mg/kg COS; and three synbiotic supplementation groups, fed with artificial crop milk + 150 mg/kg COS + 200, 300, and 400 mg/kg *C. butyricum*. The results showed that a diet supplemented with COS-*C. butyricum* synbiotic benefitted the growth performance of early-weaned squabs; even so the differences were not significant among the five groups (*p* > 0.05). In addition, dietary supplementation of 150 mg/kg COS + 300~400 mg/kg *C. butyricum* significantly improved the intestinal morphology (especially villus surface area and the ratio of villus height to crypt depth), the activity of digestive enzymes (lipase, trypsin, and leucine aminopeptidase) in duodenum contents, and the production of total short-chain fatty acids and acetic acid in ileum content (*p* < 0.05). Additionally, dietary supplementation of 150 mg/kg COS + 400 mg/kg *C. butyricum* benefitted gut health by improving the antioxidant capacity (glutathione peroxidase and total antioxidant capacity) and cytokine status (IL-4 and IL-10) (*p* < 0.05), as well as by improving the intestinal microbiota diversity. In conclusion, our results revealed that dietary supplementation with synbiotic (150 mg/kg COS + 300~400 mg/kg *C. butyricum*) could relieve early-weaned stress by maintaining intestinal health in pigeon squabs.

## Introduction

It is well-known that the pigeon belongs to altricial birds, which means that there are many differences between altricial birds and precocial birds (such as chickens, ducks, and geese). Compared with precocious birds, altrices are the most representative of their growth rate, reproductive characteristics, and access to nutrition (1, 2). Newborn squabs are always hatched with unopened eyes and reared by crop milk that is produced by both male and female pigeons (3). For that reason, pigeons are not able to feed on their own and leave their parents until they are at a market weight of 500 g (about the age of 25 days) (4). This will limit the ability of their parental pigeons to reproduce. Hence, this special stage on altricial birds including pigeons is named “weaning,” which is similar to some mammals, such as piglets and cows.

Many studies have stated that weaning, as a kind of key stage, is generally accompanied by reducing the rate of nutrition utilization, decreasing feed intake and body weight (BW), and even damaging intestinal morphology and function, which result from impaired intestinal epithelium structural and functional integrities. It is observed that growth performance is related to gut health. Published articles demonstrate that early weaning negatively impacts intestinal development and function (5–7).

On the basis of a previous study, we demonstrated that early weaning impaired intestinal development and intestinal health of pigeon squabs during the artificial rearing periods (8). When squabs are weaned at 7 days of age, their gastrointestinal tract is too immature to deal with physiological and environmental stresses induced by changes from parental feeding to artificial feeding, which consequently leads to intestinal disorder, adversely affecting the rate of survival and disease resistance of post-weaning pigeon squabs.

Variations of feed additions have been studied to be feasible options for facilitating advanced intestinal health and optimizing the weaning transition in animals. It is stated that a probiotic is a type of “live microbial feed supplement,” which means that probiotic beneficially affects animals’ growth performance by maintaining intestinal microbial balance and preventing digestive disorders (9, 10). As a widely used probiotic, *Clostridium butyricum* plays an important role in feed addition in terms of improving growth performance and benefitting intestinal health (11). It is stated that dietary supplementation with appropriate doses of *C. butyricum* could enhance digestive enzyme activity, maintain intestinal barrier function, and relieve intestinal inflammation in weaned piglets (12).

Chitosan oligosaccharides (COS), as a prebiotic, could effectively maintain intestinal structural integrity and improve intestinal immunity in early-weaned piglets (13). It is demonstrated that diet COS could enhance immune function by increasing serum IgG, IgA, IgM, and interleukin concentrations in weaned piglets (14). In addition, several studies have indicated that diet COS has a beneficial influence on growth performance by maintaining intestinal morphology and microflora, nutrition digestion, and absorption in post-weaned piglets (15–17).

Synbiotics are composed of probiotics and prebiotics. Synbiotic could improve BW, which could be attributed to promoting favorable conditions for the colonization of beneficial microflora in the gut and then improving the growth performance of broilers (18). However, knowledge about the potential effect of COS-*C. butyricum* synbiotics on early-weaned animals, especially on altricial birds, is very limited. Considering the beneficial effects of COS and *C. butyricum* on early-weaned animals, we hypothesized that dietary supplementation with COS-*C. butyricum* synbiotic could relieve early weaning stress in squabs. To test this hypothesis, we evaluated the effects of COS-*C. butyricum* synbiotic on growth performance and intestinal health in early-weaned pigeon squabs.

## Materials and Methods

### Animals and Experimental Design

In this study, the COS was purchased from Zhejiang MingZhu Animal Health Products Co., Ltd., with molecular weights at 1 kDa (Hangzhou, China). The degree of deacetylation was 85%~90%. Furthermore, 2.0 × 10^8^ cfu *C. butyricum*/g was used. The live *C. butyricum* preparation was provided by Hubei GreenSnow Biological Technology Co., Ltd. (Xianning, China).

White King Pigeon squabs (mixed sex, weaned at 7 days of age) were obtained from a commercial farm (Baixiang Pigeon Breeding Co., Ltd., Hangzhou, China). A total of 160 squabs were randomly divided into five treatments: the control group (C), fed with artificial crop milk; the COS supplementation group (CO), fed with artificial crop milk + 150 mg/kg COS; and three synbiotic supplementation groups, fed with artificial crop milk + 150 mg/kg COS + 200 (CB1), 300 (CB2), and 400 mg/kg (CB3) *C. butyricum*. Each treatment consisted of eight replications, and each replication included 4 squabs. The squabs under the same treatment were fed in one cage (50 cm width × 55 cm depth × 55 cm height) with perches and nests during the period of experiments. All birds were maintained under the same space with standardized conditions (12-h light–dark cycle) in order to reduce the error from the environment throughout the entire experimental period. The indoor relative humidity and temperature were 60%~70% and 18°C~26°C, respectively. The whole squabs were fed with artificial crop milk (17.77% protein and energy content of 13.04 MJ/kg), which was self-developed by our group. The ingredients and analyzed and calculated nutrient levels of artificial crop milk for the squabs are shown in **Table 1**. This artificial pigeon milk is a kind of powdered solid feed that was mixed with warm water (approximately 37°C) and then fed by a 100-ml syringe. The experimental squabs from these five groups were fed by hand three times a day (8:00 a.m., 12:00 p.m., and 7:00 p.m.) from 7 to 25 days of age.

**Table 1:** Ingredient composition and nutrient levels of the artificial crop milk.

Items	Content, %
Corn	40.55
Wheat	14.67
Pea	6.01
Sorghum	18.77
Soybean meal	10.00
Fish meal	1.00
Premix^1^	2.00
Canola oil	2.00
Casein	5.00
Total	100.00
Calculated nutrients^2^, % Metabolizable energy (MJ/kg)	13.04
Crude protein	17.77
Crude fat	4.69
Calcium	0.15
Total phosphorous	0.38
Analyzed nutrients, %	
Crude protein	17.89
Crude fat	4.83
Calcium	0.27
Total phosphorous	0.54

^1^The premix provided the following per kg of diet: vitamin A 2,500 IU, vitamin D_3_ 500 IU, vitamin E 2,500 IU, vitamin K_3_ 500 IU, vitamin B_1_ 500 IU, vitamin B_3_ 500 IU, CU (as copper sulfate) 10 mg, Fe (as ferrous sulfate) 100 mg, Mn (as manganese sulfate) 50 mg, and Zn (as zinc sulfate) 80 mg.

^2^Nutrition values and metabolizable energy values determined in pigeons were calculated from table of feed composition and nutritive values in China (thirty-first edition, 2020).

### Bird Slaughter and Sample Collection

During the experimental period, the rate of survival of five groups was recorded persistently. The BW of each squab was recorded at 7 and 25 days old, in order to evaluate the average daily gain (ADG). Eight squabs per treatment (one squab from each replication) were chosen for sampling at the age of 25 days after being fasted for 12 h.

The birds were killed by cervical dislocation. Small intestine samples including the duodenum, jejunum, and ileum, and their contents were dissected and collected into EP tubes. The iliac chyme samples were collected and transferred into sterile precooled tubes with ileum segments of approximately 2~3 cm and then stored at −80°C. The two sections were ligated with sterile cotton thread. All the samples were collected into sterile plastic tubes, immediately frozen in liquid N_2_, and then stored at −80°C for further analysis.

### Growth Performance and Immune Organ Index

The average BW, ADG, and the rate of survival were measured and calculated for each repetition at 25 days of age. The weight of the immune organ samples including the thymus gland, spleen, and bursa was dissected from the squabs with the surrounding fat removed and weighed, and then the weight of each immune organ from the squabs was recorded in order to calculate the immune organ index. Immune organ index (g/kg) = immune organ weight (g)/live body weight (kg).

### Small Intestinal Morphometric Traits

Small intestine samples of approximately 1 cm were obtained from the duodenum, jejunum, and ileum, flushed with normal saline, and stored in a 4% neutral-buffered formalin solution for histopathology for at least 24 h. These segments of the small intestine were dehydrated and embedded in paraffin, deparaffinized in xylene, rehydrated in a graded alcohol series, examined by light microscopy (Mshot, MF52, Guangzhou, China), and analyzed with Mshot Image Analysis System 1.1.4 software (Mshot Corporation, Guangzhou, China). Villus height was defined as the distance from the tip of the villi to the villus crypt junction. The depth of the invagination between adjacent villi was called crypt depth. The surface area of each villus could be calculated by the formula [(2π) × (villus width / 2) × (villus height)] (19).The ratio of villus height to crypt depth (VCR) was also calculated. Each replicate per segment recorded information from at least 30 villi or crypts (20).

### Determination of Intestinal Enzymatic Activity

The samples of contents from the duodenum were stored at −80°C in order to measure digestive enzyme activity. The tissue homogenate was comprised of ice-cold phosphate-buffered saline (PBS) (pH 7.0) and a duodenum content sample at 9:1 at 4°C. The homogenates were centrifuged at 2,500 rpm for 10 min, and then the supernatant was collected and used for testing.

The activities of lipase (LPS), trypsin, α-amylase, maltase, sucrase, aminopeptidase-N (APN), and leucine aminopeptidase (LAP) were assessed in this study. The activity of APN and LAP was measured by ELISA kits at 450 nm (Shanghai Enzyme-linked Biotechnology Co., Ltd., Shanghai, China). The rest of the five enzymes’ activity was measured using commercial assay kits (Nanjing Jiancheng Bioengineering Institute, Nanjing, China).

The total protein (TP) concentration of duodenum contents was determined by assay kits and was used for calculating the intensity of these seven digestive enzymes’ activity. The application solution of Coomassie Brilliant Blue was comprised of distilled water and Coomassie Brilliant Blue stock solution at 4:1 and followed the instruction to measure the different samples’ OD values at 595 nm, and then the intensity of different digestive enzyme activities was calculated following the formula from instruction.

One lipase activity unit of duodenum contents is defined as 1 μmol of substrate consumed per minute per gram of tissue protein at 37°C. One activity of the trypsin unit is defined as every 0.003 change in absorbance per minute caused by trypsin in 1 mg protein from duodenal chyme. This reaction system is controlled at 37°C, pH = 8.0. According to a previous study, α-amylase activity was determined (21). One α-amylase activity unit was defined as 10 mg of starch hydrolyzed within 30 min with an enzyme in 1 mg of tissue protein at 37°C. One maltase or sucrase activity unit was defined as 1 mmol of maltase or sucrose hydrolyzed by 1 mg of tissue protein for 1 min at 37°C, pH 6.0, reaction system. Enzyme activity of APN and LAP was calculated with the standard curve. One unit of APN and LAP in duodenum chyme was expressed as 1 ng of standard antibody per milliliter solution measured at 37°C.

### Jejunal Antioxidant Status and Cytokine Analysis

About 0.1 g of jejunum tissue sample was homogenized at a ratio of 1:9 (weight/volume) with ice-cold PBS at 4°C. The homogenates were centrifuged at 2,500 rpm for 10 min, and then the supernatant was collected and used for measuring the determination of jejunal tissue oxidative status and cytokines. The TP concentration was determined following the same methods mentioned above. The activities of glutathione peroxidase (GSH-Px), total superoxide dismutase (T-SOD) and catalase (CAT), the concentration of malondialdehyde (MDA), and the total antioxidant capacity (T-AOC) were determined according to the manufacturer’s instructions accompanying the assay kit (Nanjing Jiancheng Bioengineering Institute, Nanjing, China).

The concentrations of tumor necrosis factor alpha (TNF-α), tumor necrosis factor beta (TNF-β), gamma interferon (IFN-γ), interleukin 2 (IL-2), IL-4, IL-6, IL-10, and IL-1β in jejunal segments were determined by absorbance changes at 450 nm with ELISA kits (Shanghai Enzyme-linked Biotechnology Co., Ltd., Shanghai, China) according to the manufacturer’s protocol. Concentrations of cytokine were calculated with the standard curve.

### Ileal Short-Chain Fatty Acid Extraction and Analysis

About 0.80 ± 0.01 g of ileal contents were placed into a sterilized tube for preparing 10% suspension (22). The supernatant (approximately 500 µl) was transferred into a 1.5-ml tube containing 100 µl of crotonic acid–metaphosphoric acid. The mixture was incubated for 24 h at −30°C, and then the mixture was centrifuged at 8,000 rpm, at 4°C for 3 min to remove the impurities such as protein. A 0.22-µm organic filter was used to filter the liquid (0.5 ml) into gas chromatography (GC) tubes for the determination of short-chain fatty acids (SCFAs) using GC (Agilent 7890A, California, USA). The SCFAs were analyzed using a fused-silica capillary column, 30 m × 0.25 mm ID × 0.25 μm. The temperatures of the FID and injector were 250°C and 220°C, respectively, and the column was gradually heated from 80°C to 170°C at a rate of 20°C min^−1^ and held for 1 min.

### Ileal Microbial DNA Extraction and 16S rDNA Sequencing

The entire ileal contents were collected and used for bacterial 16S rDNA sequencing. Bacterial genomic DNA was extracted from a 0.1-g sample using the cetyltrimethylammonium bromide/sodium dodecyl sulfate (CTAB/SDS) method. The V3–V4 hypervariable regions of the bacterial 16S rRNA gene were amplified using primers 341 F: 5′-CCTAYGGGRBGCASCAG-3′ and 806 R: 5′-GGACTACNNGGGTATCTAAT-3′. 16S rDNA genes were amplified starting from about 10 ng of template DNA in a reaction system with 0.2 μM of forward and reverse primers. All PCRs were carried out following cycles conditions: an initial denaturation at 98°C for 1 min, followed by 30 cycles of denaturation at 98°C for 10 s, annealing at 50°C for 30 s, and elongation at 72°C for 1 min, for an extension step at 72°C for 5 min in the end. The products were visualized by electrophoresis on 2% agarose gel. Samples with a bright main strip between 400 and 450 bp were prepared for the later tests. Then, mixed PCR products were performed in a purification step with AxyPrepDNA Gel Extraction Kit (Axygen Scientific, Inc., Union City, CA, USA). Finally, the library was indexed on an Illumina Miseq/HiSeq2500 platform.

The clean sequences were assigned to the same operational taxonomic units (OTUs) with more than 97% similarity. According to the OTU tables, alpha diversity could be calculated such as Chao1, Shannon index, and observed species. Cluster analysis was preceded by non-metric multidimensional scaling (NMDS) analysis based on weighted_unifrac distance, which was applied to reduce the dimension of the original variables using the QIIME software package. To identify differences in microbial communities among the five groups, an analysis of similarities (ANOSIM) was conducted based on the abundance of OTUs.

### Statistical Analyses

Statistical analysis was performed using GraphPad Prism statistical software (GraphPad Software 8.0.2 Inc., La Jolla, CA, USA). The results are shown as figures, which were also generated using GraphPad Prism 8.0.2 software. The significant differences among these five treatment means were compared by Tukey’s multiple-comparison test following one-way ANOVA. The final data presented were shown as the means with SEM as plotting, the values considered significant at *p*-value were less than 0.05, and very significant differences were considered at *p* < 0.01.

## Results

### Growth Performance and Immune Organ Index

**Figure 1:**
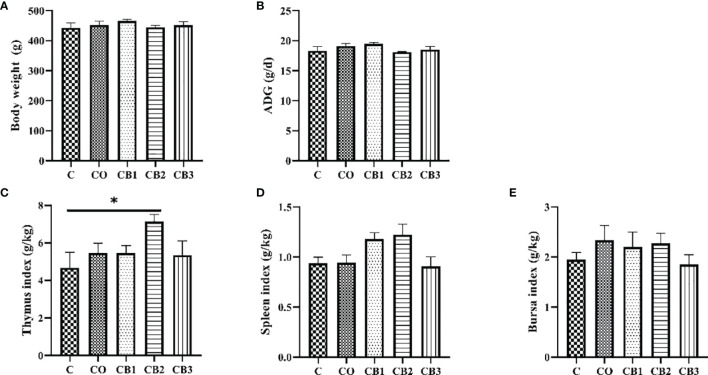
Effects of dietary chitosan oligosaccharides (COS) and *Clostridium butyricum* synbiotic supplementation on growth performance and immune organ index in early-weaned pigeon squabs. **(A)** Body weight of squabs at 25 days of age. **(B)** ADG, average daily gain. **(C)** Thymus index. **(D)** Spleen index. **(E)** Bursa index. Values are means with the SEM of eight squabs. *p < 0.05. C group, control group, squabs fed with artificial crop milk; CO group, squabs fed with artificial crop milk + 150 mg/kg COS; CB1, CB2, and CB3 group, squabs fed with artificial crop milk + 150 mg/kg COS + 200, 300, and 400 mg/kg *C. butyricum*, respectively.

The effect of dietary COS-*C. butyricum* synbiotic supplementation on growth performance including BW and ADG, and immune organ index including thymus index, spleen index, and bursa index of early-weaned squabs are shown in **Figures 1A-E**. The results show that dietary COS-*C. butyricum* synbiotic benefits BW and ADG in early weaning squabs, but there are no significant difference changes among these five groups (**Figures 1A,B**; *p* > 0.05). In addition, a numeric increase (*p* > 0.05) in survival rate for squabs was observed in response to dietary supplementation with COS-*C. butyricum* synbiotic, i.e., from 88.9% in the C group to 90%~95% in the synbiotic supplementation groups. As compared with the C group, dietary COS-*C. butyricum* synbiotic led to an increase in immune organ index of Day 25 squabs in the thymus (**Figure 1C**). The thymus index of squabs in the CB2 groups increased significantly (*p* < 0.05). A slight increase was presented in **Figures 1D,E** on the spleen index and bursa index, but there was no significant difference (*p* > 0.05).

### Small Intestinal Morphology Examination

**Figure 2:**
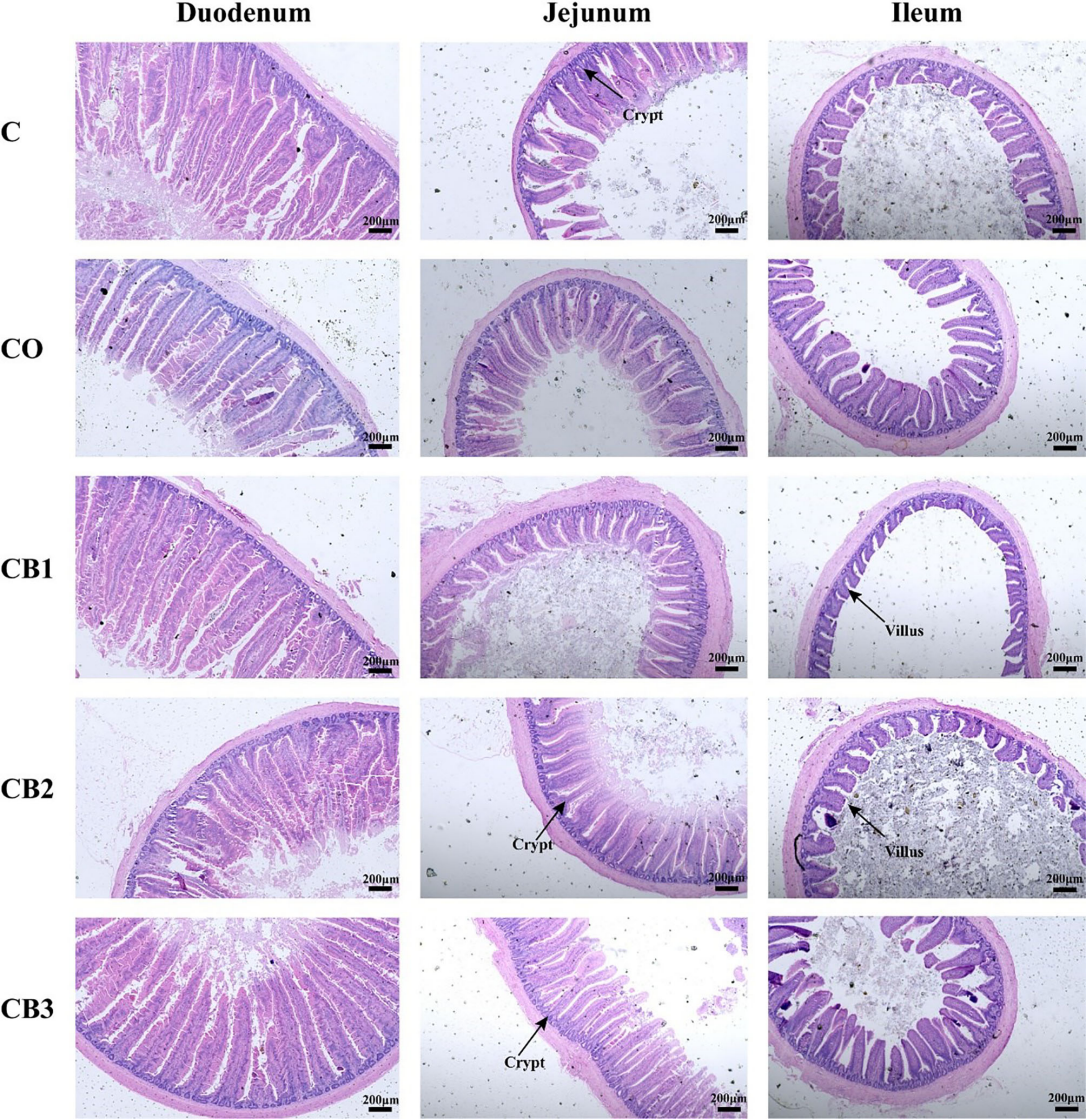
Effects of dietary chitosan oligosaccharides (COS) and *Clostridium butyricum* synbiotic supplementation on intestinal morphology of duodenum, jejunum, and ileum in early-weaned pigeon squabs. Sections were stained with H&E. C group, control group, squabs fed with artificial crop milk; CO group, squabs fed with artificial crop milk + 150 mg/kg COS; CB1, CB2, and CB3 group, squabs fed with artificial crop milk + 150 mg/kg COS + 200, 300, and 400 mg/kg *C. butyricum*, respectively.

**Figure 3:**
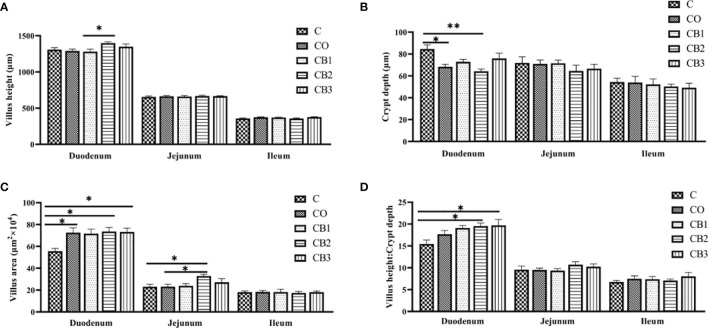
Effects of dietary chitosan oligosaccharides (COS) and *Clostridium butyricum* synbiotic supplementation on small intestine morphometric traits of three segments (duodenum, jejunum, and ileum) in early-weaned pigeon squabs. **(A)** Villus height. **(B)** Crypt depth. **(C)** Villus area. **(D)** The ratio of villus height to crypt depth. Values are means with the SEM of eight squabs. *p < 0.05 and **p < 0.01. C group, control group, squabs fed with artificial crop milk; CO group, squabs fed with artificial crop milk + 150 mg/kg COS; CB1, CB2, and CB3 group, squabs fed with artificial crop milk + 150 mg/kg COS + 200, 300, and 400 mg/kg *C. butyricum*, respectively.

H&E staining of the small intestines (duodenum, jejunum, and ileum) of the five groups is shown in **Figure 2**. Compared with the C group, the duodenum, jejunum, and ileum of squabs in the CO group revealed a normal appearance with the regular and complete intestinal structure of the villus and crypt. A better visual structural integrity of the duodenum, jejunum, and ileum in the CB1, CB2, and CB3 groups than in the C group is shown. In the duodenum, the villus area and VCR in the CB3 group were significantly higher (*p* < 0.05) than those in the C group (**Figures 3C,D**). Compared with the C group, crypt depth decreased significantly (*p* < 0.01) in the CB2 group (**Figure 3B**), while VCR increased significantly (*p* < 0.05) in the CB2 and CB3 groups (**Figure 3D**). In the jejunum, there was no significant difference among the five groups in villus height, crypt depth, and VCR. Compared with the C group, the villus area in the CB2 group increased significantly (*p* < 0.05) (**Figure 3C**). The villus morphology in the ileum of squabs among these five groups did not differ significantly (**Figures 3A-D**; *p* > 0.05).

### Determination of Enzymatic Activity

**Figure 4:**
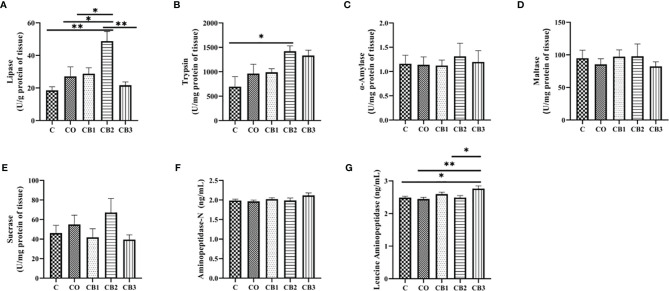
Effects of dietary chitosan oligosaccharides (COS) and *Clostridium butyricum* synbiotic supplementation on digestive enzyme activity of duodenum content in early-weaned pigeon squabs. **(A)** Lipase. **(B)** Trypsin. **(C)** α-Amylase. **(D)** Maltase. **(E)** Sucrase. **(F)** Aminopeptidase-N. **(G)** Leucine aminopeptidase. Values are means with the SEM of eight squabs. *p < 0.05 and **p < 0.01. C group, control group, squabs fed with artificial crop milk; CO group, squabs fed with artificial crop milk + 150 mg/kg COS; CB1, CB2, and CB3 group, squabs fed with artificial crop milk + 150 mg/kg COS + 200, 300, and 400 mg/kg *C. butyricum*, respectively.

The results of the effect of COS-*C. butyricum* synbiotic on digestive enzyme activity in duodenum content in early-weaned pigeons are presented in **Figures 4A-G**. Compared with the C group, the activity of lipase (**Figure 4A**) and trypsin (**Figure 4B**) increased significantly (*p* < 0.05) in the CB2 group. Squabs in the CB3 group had a higher (*p* < 0.05) activity of LAP (**Figure 4G**).

### Determination of Intestinal Mucosal Oxidant Status

**Figure 5:**
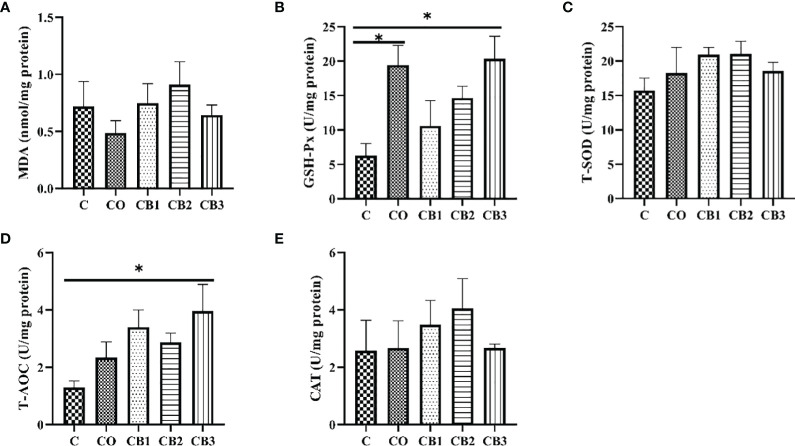
Effects of dietary chitosan oligosaccharides (COS) and *Clostridium butyricum* synbiotic supplementation on the antioxidant status of jejunum mucosa in early-weaned pigeon squabs. **(A)** MDA, malondialdehyde. **(B)** GSH-Px, glutathione peroxidase. **(C)** T-SOD, total superoxide dismutase. **(D)** T-AOC, total antioxidant capacity. **(E)** CAT, catalase. Values are means with the SEM of eight squabs. *p < 0.*05*. C group, control group, squabs fed with artificial crop milk; CO group, squabs fed with artificial crop milk + 150 mg/kg COS; CB1, CB2, and CB3 group, squabs fed with artificial crop milk + 150 mg/kg COS + 200, 300, and 400 mg/kg *C. butyricum*, respectively.

The effect of dietary COS-*C. butyricum* synbiotic supplementation on the antioxidant status of jejunum mucosa is given in **Figures 5A-E**. Compared with the C group, the activity of GSH-Px increased significantly (*p* < 0.05) in both the CO and CB3 groups (**Figure 5B**). A significant increase (*p* < 0.05) was observed for T-AOC in the CB3 group in comparison with the C group (**Figure 5D**).

### Jejunal Inflammatory Cytokine Analysis

**Figure 6:**
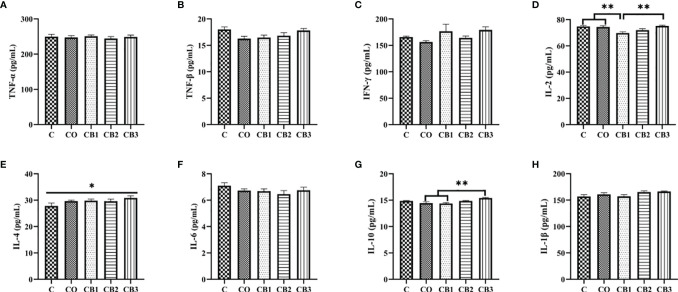
Effects of dietary chitosan oligosaccharides (COS) and *Clostridium butyricum* synbiotic supplementation on inflammatory cytokines of jejunum in early-weaned pigeon squabs. **(A)** TNF-α, tumor necrosis factor alpha. **(B)** TNF-β, tumor necrosis factor beta. **(C)** IFN-γ, gamma interferon. **(D)** IL-2, interleukin 2. **(E)** IL-4. **(F)** IL-6. **(G)** IL-10. **(H)** IL-1β. Values are means with the SEM of eight squabs. *p < 0.05 and **p < 0.01. C group, control group, squabs fed with artificial crop milk; CO group, squabs fed with artificial crop milk + 150 mg/kg COS; CB1, CB2, and CB3 group, squabs fed with artificial crop milk + 150 mg/kg COS + 200, 300, and 400 mg/kg C. *butyricum*, respectively.

The influence of dietary COS-*C. butyricum* synbiotic supplementation on the levels of jejunum cytokines is summarized in **Figures 6A-H**. Squabs in the CB1 group expressed a higher (*p* < 0.05) level of IL-2 than in the C and CO groups (**Figure 6D**). Squabs in the CB3 group expressed a higher (*p* < 0.05) level of IL-4 than in the C group (**Figure 6E**). The level of IL-10 was much higher (*p* < 0.01) in the CB3 group than in the CO and CB1 groups (**Figure 6G**).

### Ileal Short-Chain Fatty Acid Production and Microbiota Analysis

As mentioned above, dietary COS-*C. butyricum* synbiotic supplementation in the CB2 or CB3 group significantly improved small intestine morphometric traits, digestive enzyme activity, antioxidant capacity, and cytokine status. To better understand the effects of COS-*C. butyricum* synbiotic supplementation on intestinal health, the changes in SCFAs production, and microbiota composition in the CB2 and CB3 groups in comparison with the C group were further analyzed.

**Figure 7:**
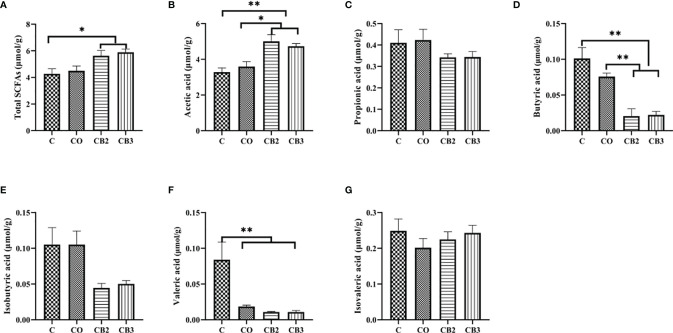
Effects of dietary chitosan oligosaccharides (COS) and *Clostridium butyricum* synbiotic supplementation on the concentrations of short-chain fatty acids (SCFAs) in ileum content of early-weaned pigeon squabs. **(A)** Total SCFAs. **(B)** Acetic acid. **(C)** Propionic acid. **(D)** Butyric acid. **(E)** Isobutyric acid. **(F)** Valeric acid. **(G)** Isovaleric acid. Values are means with the SEM of eight squabs. *p < 0.05 and **p < 0.01. C group, control group, squabs fed with artificial crop milk; CO group, squabs fed with artificial crop milk + 150 mg/kg COS; CB2 group, squabs fed with artificial crop milk + 150 mg/kg COS + 300 mg/kg *C. butyricum*.

**Figures 7A-G** show the influence of dietary COS-*C. butyricum* synbiotic supplementation on levels of SCFAs in ileal contents. The total SCFA concentration increased (*p* < 0.05) in the CB2 and CB3 groups significantly compared with the C group (**Figure 7A**). Simultaneously, the concentration of acetic acid in the CB2 and CB3 groups was much higher (*p* < 0.05) than in the C and CO groups (**Figure 7B**). However, the level of butyric acid decreased significantly (*p* < 0.01) in the CB2 and CB3 groups in comparison with the C and CO groups (**Figure 7D**). Compared with the C group, a significant decrease (*p* < 0.01) was observed in valeric acid in the CB2 and CB3 groups (**Figure 7F**).

**Figure 8:**
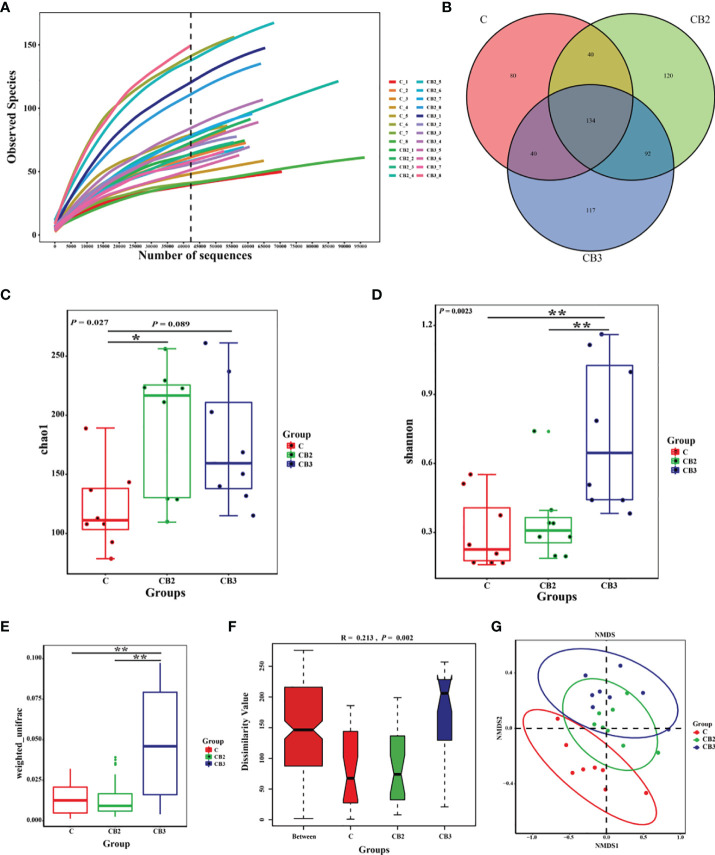
Effects of dietary chitosan oligosaccharides (COS) and *Clostridium butyricum* synbiotic supplementation on ileal microbiota in early-weaned pigeon squabs. **(A)** Rarefaction curves. **(B)** Venn diagram of operational taxonomic units (OTUs). **(C)** Microbiota richness is represented by Chao1 index. **(D)** Microbiota diversity is represented by Shannon index. **(E)** Weighted_unifrac distance. **(F)** Analysis of similarities based on OTUs. **(G)** Non-metric multidimensional scaling (NMDS) analysis plot of bacterial communities. Values are means with the SEM of eight squabs. *p < 0.05 and **p < 0.01. C group, control group, squabs fed with artificial crop milk; CO group, squabs fed with artificial crop milk + 150 mg/kg COS; CB2 and CB3 group, squabs fed with artificial crop milk + 150 mg/kg COS + 300 and 400 mg/kg C *butyricum*, respectively.

**Figure 9:**
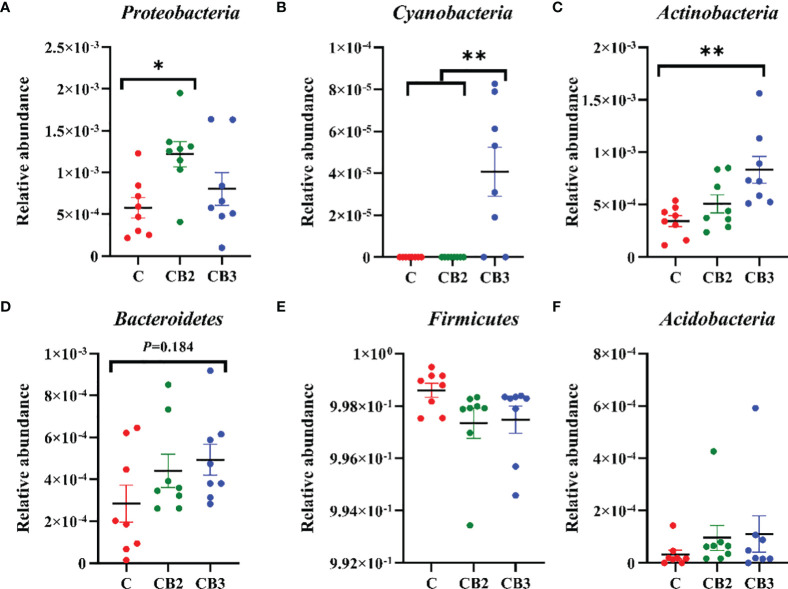
Effects of dietary chitosan oligosaccharides (COS) and *Clostridium butyricum* synbiotic supplementation on the relative abundance of ileal microbiota at the phylum level in early-weaned pigeon squabs. **(A)** Proteobacteria. **(B)** Cyanobacteria. **(C)** Actinobacteria. **(D)** Bacteroidetes. **(E)** Firmicutes. **(F)** Acidobacteria. Values are means with the SEM of eight squabs. *p < 0.05 and **p < 0.01. C group, control group, squabs fed with artificial crop milk; CO group, squabs fed with artificial crop milk + 150 mg/kg COS; CB2 and CB3 group, squabs fed with artificial crop milk + 150 mg/kg COS + 300 and 400 mg/kg *C. butyricum*, respectively.

**Figure 10:**
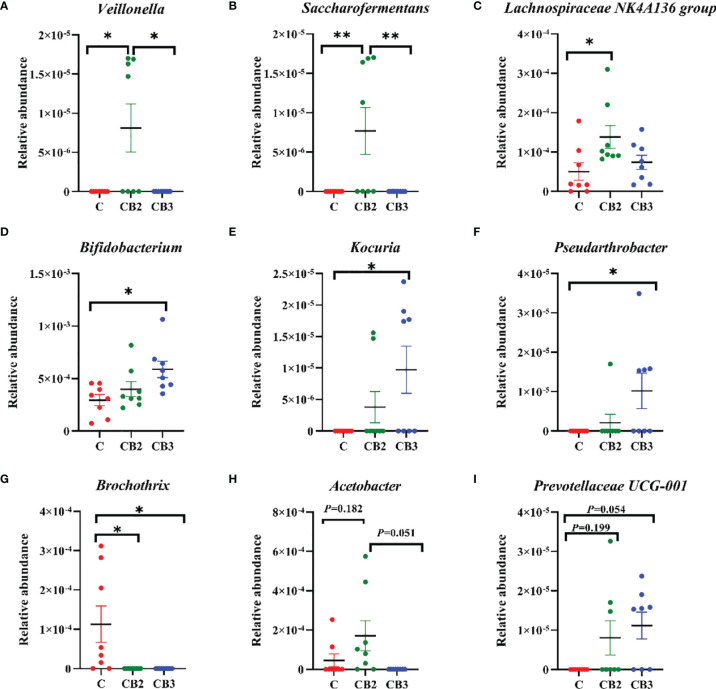
Effects of dietary chitosan oligosaccharides (COS) and *Clostridium butyricum* synbiotic supplementation on the relative abundance of ileal microbiota at the genus level in early-weaned pigeon squabs. **(A)** *Veillonella*. **(B)** *Saccharofermentans*. **(C)** *Lachnospiraceae NK4A136 group*. **(D)** *Bifidobacterium*. **(E)** *Kocuria*. **(F)** *Pseudarthrobacter*. **(G)** *Brochothrix*. **(H)** *Acetobacter*. **(I)** *Prevotellaceae UCG-001*. Values are means with the SEM of eight squabs. *p < 0.05 and **p < 0.01. C group, control group, squabs fed with artificial crop milk; CO group, squabs fed with artificial crop milk + 150 mg/kg COS; CB2 and CB3 group, squabs fed with artificial crop milk + 150 mg/kg COS + 300 and 400 mg/kg *C. butyricum*, respectively.

The abundance and diversity of ileal microorganisms were obtained using data from 16S rRNA high-throughput sequencing on squabs at 25 days of age. The rarefaction curve of observed species plateaued when the read increased to a certain level revealing that there was sufficient OUT coverage to accurately describe the bacterial composition of each group (**Figure 8A**). The Venn diagram in **Figure 8B** shows that the squabs in the C, CB2, and CB3 groups had 80, 120, and 117 unique OTUs, respectively. The CB2 group had significantly higher (*p* < 0.05) microbiota richness than the C group (**Figure 8C**). The Shannon α-diversity index in the CB3 group was significantly higher (*p* < 0.01) than in both the C and CB2 groups (**Figure 8D**). ANOSIM and NMDS indicated that there was no noticeable separation between the C and CB2 groups, but the CB3 group was clearly separated from the C group (**Figures 8E-G**). At the phylum level (**Figures 9A-F**), the results showed that the relative abundance of Proteobacteria in the CB2 group increased significantly (*p* < 0.05) compared with that in the C group, while the relative abundance of Cyanobacteria and Actinobacteria in the CB3 group increased significantly (*p* < 0.01) compared with those in both the C and CB2 group (**Figures 9A-C**). On the genus level (**Figures 10A-I**), *Veillonella* (*p* < 0.05), *Saccharofermentans* (*p* < 0.01), and *Lachnospiraceae NK4A136 group* (*p* < 0.05) showed the highest abundances in the CB2 group (**Figures 10A-C**), while *Bifidobacterium*, *Kocuria*, and *Pseudarthrobacter* showed the highest (*p* < 0.05) abundances in the CB3 group (**Figures 10D-F**). The relative abundance of *Brochothrix* increased (*p* < 0.05) in both the CB2 and CB3 groups significantly compared with the C group (**Figure 10G**).

## Discussion

With the increasing requirements for pigeon meat in the market in southern China and even in Southeast Asia, pigeons have been reared as a kind of poultry like ducks and broilers (23). As we all know, much attention has been paid to the nutrition levels and delicacy of meat from pigeons. Furthermore, studies have reported that pigeons have been observed for their profitable sources of meat from *Columba* species since they are always growing and developing rather more quickly with less input (24). High growth performance plays an essential and necessary role in the poultry industry, especially in meat poultry like broilers, ducks, and pigeons as well (25).

In previous studies, we found that early weaning did harm the growth performance of squabs at the age of 25 days. Considering the potential benefits of the probiotics and prebiotics in intestine health, we are willing to attempt to relieve the weaning stress on post-weaned young pigeons with the feed addition of COS-*C. butyricum* synbiotic. In this research, a diet with COS and *C. butyricum*, either alone or in combination, had no significant effects on growth performance, but numerically increased the BW, ADG, and survival rates of early-weaned pigeon squabs. Similar to our results, it is stated that there was no significant difference in the growth performance in broilers when *C. butyricum* was added in dietary at a level of 1 × 10^9^ cfu/kg (26). In addition, a similar result was also observed in published studies that have reported that supplementation with *C. butyricum* did not have any effects on growth performance in post-weaning piglets (27, 28). COS, as a kind of prebiotics, was attempted in sows and weaning piglets in a previous study and increased the growth performance such as BW and ADG in post-weaned piglets (13). The higher the weight of the animal is, the more energy the animal needs. The magnitude of variation in weight in organs across changing physiological environments means energetic high requirements (29). The immune organ index is always considered an essential reference for the growth and development of the immune level of poultry since immune organs (thymus, spleen, and bursa) are important for poultry to reflect the functional state and development of the organism to a certain extent. In this study, a significant increase could be observed in the thymus index in the CB2 group in comparison with the C group. Based on our observations in BW, ADG, survival rate, and immune organ index, as well as the given role of COS and *C. butyricum* in animals, it is supposed that dietary COS-*C. butyricum* synbiotic could have a certain role in benefiting growth performance in early-weaned pigeon squabs. Because this study is the first attempt to explore dietary *C. butyricum* in combination with COS, further studies are needed to verify whether the COS-*C. butyricum* synbiotic could improve growth performance in other weaned animals.

It is well-known that improved growth performance is always related to the ability of the digestive and absorption of the small intestine. We previously found that early weaning did harm the integrity and function of the villus in the small intestine of squabs (8). In addition, mounting evidence has also indicated that early weaning-induced stress had adverse effects on intestinal epithelial morphology traits in newborn piglets and was harmful to health and feed utilization efficiency in swine (30, 31). The villus contributes most of the luminal surface in the intestine, which is critical for the efficiency of absorption (32). Villus height, crypt depth, and surface area of villus are measured in most studies in order to determine the structure of the intestinal villus. Previous studies suggested that probiotics and prebiotics are able to enhance villous height and make crypt depth lower in the small intestine, which is beneficial for the digestibility and absorption of nutrition. In this study, we found that the morphometric traits were improved in COS-*C. butyricum* synbiotic supplementation groups (CB2 and CB3), and the changes of duodenal villi are more obvious than in the other two small intestinal segments (jejunum and ileum). Studies have indicated that higher villous height and lower crypt depth could be observed in mice and post-weaning piglets with the dietary *C. butyricum* supplementation (33, 34). Furthermore, some studies have proven that dietary COS could improve villus morphology and structure of the small intestine in broilers and weaned piglets (35, 36).

The morphological changes observed in the small intestine around weaning are closely related to changes in the mucosal enzyme activity observed at the same time (37). As mentioned above, we found that the morphology of the small intestinal villus in the duodenum changed significantly. It is declared that changed small intestinal villous morphology would affect the ability of digestion and absorption of nutrients, which also had a potential effect on the activity of digestion enzymes in the small intestine. Studies showed that the differences in enterocyte life span were able to lead to differences in digestive enzyme activity and in the crypt-villus enzyme activity gradient between the proximal and distal segments as well (38). Moreover, it is apparent that increased digestive enzyme activity is related to the differentiation of enterocytes for migrating from the crypt to its villus (39). We observed that the activity of lipase, trypsin, and LAP in COS-*C. butyricum* synbiotic supplementation groups (CB2 and CB3) increased significantly as compared with the C group in this study. Many studies have stated that *C. butyricum* was beneficial for the lipase and trypsin in weaned piglets, rabbits, and other poultry such as broilers (12, 40, 41). These results may arise from the probability that the probiotic itself could produce digestive enzymes; in order to adapt to this supplementation, the squabs also enhance the secretion of this type of digestion. In addition, studies showed that *C. butyricum* would decrease the pH in the small intestine environment, in which the digestive enzyme activity may be increased and then benefit the absorption of nutrients. Beyond that, combined with the characteristics of pigeons, the higher lipase activity may be related to the digestion and absorption function of squabs. Unlike mammals and other domestic birds, squabs are fed by parental pigeons with crop milk comprised of mainly proteins and lipids (42, 43). Therefore, we speculated that adding an appropriate amount of *C. butyricum* can effectively improve the digestion and absorption capacity of squabs and is more conducive to the increase of lipase and protease expression in the digestive tract of squabs.

Lower antioxidant capacity in the small intestine is probably related to the damaged villus structure in post-weaned squabs likewise. A large number of reports have confirmed that the intestinal epithelium tends to undergo oxidative damage induced by reactive oxygen species (ROS) generated by luminal oxidants, which affects gut health and antioxidant function in the small intestine (44, 45). Additionally, the stress from early weaning could disrupt the balance of the antioxidant system and even weaken antioxidant capacities (46, 47). Numerous researchers have declared that the segment of jejunum is one of the most essential organs for oxidative stress, which would bring about impaired intestine barrier and function (48–50). Antioxidant enzymes can limit ROS to counteract oxidative stress and hinder or repair oxidative damage (51). In our experiment, we found that a higher level of GSH-Px and T-AOC could be observed in the COS-*C. butyricum* synbiotic supplementation group (CB3). Some scientists declared that *C. butyricum* could produce both butyrate and H_2_ to modulate oxidative stress by increasing antioxidant enzyme activity and reducing reactive oxygen metabolites (52). Moreover, COS could improve the oxidative capacity of the small intestine in broilers and weaned piglets (28, 35, 36).

Cytokines, which regulate the inflammatory response, play a central role in protecting the gastrointestinal environment and maintaining the balance in the small intestine in order to enhance the capacity of immune function (53). The proinflammatory cytokines, such as TNF-α, INF-γ, and IL-2, perform the function of the inflammatory response. On the contrary, IL-4 and IL-10 belong to anti-inflammatory cytokines owing to their function in the containment of inflammatory responses, protecting the host from tissue damage during acute phases of the immune response as well (54). In our study, the results demonstrated that dietary COS in combination with high-dose *C. butyricum* supplementation (CB3 group) significantly increased IL-4 and IL-10 production. Similarly, with published studies, the positive influence of *C. butyricum* and COS on anti-inflammatory cytokines was also observed in broilers and weaned mammals (11, 41). It is stated that *C. butyricum* could induce IL-10-producing macrophages *via* the TLR2/MyD88 pathway directly. IL-10, as one of the most efficient anti-inflammatory cytokines, has been proven to play an important role in regulating intestinal homeostasis during host defense in many animals (55). Furthermore, IL-10 production from macrophages in the small intestine is necessary for protecting intestinal health from colitis by means of *C. butyricum*. These results could explain that *C. butyricum* benefits jejunal immunity function by inducing the proliferation of macrophage, which has the ability to produce IL-10 (56).

It is well-known that there is a strong relationship between intestinal immune function, volatile fatty acids, and intestinal microbes (34). To further understand the benefits and role of COS-*C. butyricum* synbiotic supplementation in intestinal health, we analyzed the production of SCFAs in the ileum content of squabs. The results showed that higher concentrations of total SCFAs and acetic acid were observed in the CB2 and CB3 groups. It has been demonstrated that *C. butyricum* could increase the concentration of intestinal acetic acid in early-weaned piglets, which can enhance the capacity for digestion and absorption of nutrients. According to the reports on the function of SCFAs on humans, SCFAs may offer persistently energy and nutrition to epithelial cells in the gastrointestinal tract and hence promote appetite and intestinal motility of the hosts (57, 58). Moreover, previous research has proven that acetate induced the secretion of ghrelin, which could enhance food intake and obesity by stimulating the nervous system (59). In addition to the function of promoting digestion and absorption, acetic acid also plays an essential role in the immunity function of the host. It is declared that acetate could coordinate the interactions between epithelial and immune cells within the gut mechanistically and regulate IgA production to maintain mucosal homeostasis (60). Another interesting finding of this study is that the production of butyric acid in the CB2 and CB3 groups was decreased as compared with the C group. Published articles showed that the level of butyrate is a sort of double-edged sword for gut health in animals, sometimes referred to as the “butyrate paradox,” whereby it induces proliferation in the healthy intestine but apoptosis in transformed cells (61). Butyrate appears to be one of the most necessary regulators of tight junction protein. It has been pointed out that butyrate could enhance intestinal barrier function through increased expression of claudin-1 and zonula occludens-1, which are regarded as components of the tight junction (62). Nevertheless, there are still reports showing that a lower concentration of butyrate is also beneficial for the intestine epithelial cell cycle. Most studies showed the results of the function of butyrate in the colon of mammals instead of birds. That is to say, we could not ignore the difference in the intestine in the development and evolution between birds and mammals.

SCFAs are the primary end products of microflora in the intestine of animals (63). Meanwhile, dietary substances are directly related to the composition of intestinal microflora (64). In view of the analysis of the gut flora, much higher microflora diversity could be observed in the CB3 group. This is in accordance with the findings of previous studies (34, 65, 66). Furthermore, it is demonstrated that *C. butyricum* administered to breast-feeding maternal mice could raise the diversity of the gut flora in their offspring (67). To our knowledge, the higher the microbiota diversity, the stronger the intestine. This is because microbiota diversity is one of the prime determinants of colonization resistance against invading pathogens (68). On the genus level, we found that the relative abundance of *Bifidobacterium* in the CB2 group is higher than it in the C group, although there was no significant difference between the two groups. Previous studies in neonatal mice also showed that supplementation with *C. butyricum* regulated the balance of the intestinal flora, manifesting in increased colony counts of *Bifidobacterium* (67). Moreover, the study demonstrated a negative relationship between the concentrations of butyric acid in ileum chyme and the relative abundance of *Bifidobacterium*. It is proposed that the protective role of *Bifidobacterium* in the ileum is associated with a dramatic decrease or a disappearance in butyric acid, which is potentially deleterious in young animals (69, 70). In addition, we also found that the relative abundance of *Veillonella*, *Lachnospiraceae NK4A136 group*, and *Saccharofermentans* in the CB2 group was significantly increased. Unlike other anaerobes, *Veillonella* harbors a complete pathway for the metabolism of lactate through the tricarboxylic acid cycle to produce propionate. Propionate can increase energy expenditure in individuals, even in a fasting state (71). A previous study highlighted that the improved barrier function and metabolism phenotype observed following spermidine treatment might be associated with increased *Lachnospiraceae NK4A136 group* levels (72). *Saccharofermentans* can ferment hexoses and polysaccharides to produce acetate, lactate, and fumarate to adjust the environment in the intestine of animals and aid digestion and absorption (73). The findings mentioned above may partly explain the higher level of total SCFAs and acetic acid in the ileal chyme of early-weaned pigeons in response to dietary COS-*C. butyricum* synbiotic supplementation.

## Conclusions

In conclusion, our observations indicated that dietary COS-*C. butyricum* synbiotic supplementation could play a by-no-means negligible role in improving growth performance and intestinal health of early-weaned pigeon squabs. In addition, different proportions of a couple of COS and *C. butyricum* have various beneficial effects on the gut health of early-weaned squabs. In the current study, it is recommended that the appropriate level of COS-*C. butyricum* synbiotic supplementation should be 150 mg/kg COS + 300~400 mg/kg *C. butyricum*. However, knowledge of the beneficial role of COS-*C. butyricum* synbiotics on relieving early weaning stress appears limited; further studies are needed to verify whether the COS-*C. butyricum* synbiotic could improve growth performance and intestinal health in other early-weaned animals.

## Data Availability Statement

The datasets presented in this study can be found in online repositories. The names of the repository/repositories and accession number(s) can be found below: https://www.ncbi.nlm.nih.gov/search/all/?term=PRJNA828279.

## Ethics Statement

The animal study was reviewed and approved by Animal Care and Welfare Committee of Animal Science College and the scientific Ethical Committee of Zhejiang University (NO. ZJU2013105002) (Hangzhou, China).

## Author Contributions

XD designed the experiments. JW and WZ performed the experiments and carried out the data summarizing. JW and JL were mainly responsible for experimental animal feeding and result analysis. XD, JW, CH, and XZ wrote and revised the main manuscript. All authors read and approved the final manuscript.

## Funding

This work was supported by the National Natural Science Foundation of China (31902173) and the Natural Science Foundation of Zhejiang Province (LY22C170002).

## Conflict of Interest

The authors declare that the research was conducted in the absence of any commercial or financial relationships that could be construed as a potential conflict of interest.

## Publisher’s Note

All claims expressed in this article are solely those of the authors and do not necessarily represent those of their affiliated organizations, or those of the publisher, the editors and the reviewers. Any product that may be evaluated in this article, or claim that may be made by its manufacturer, is not guaranteed or endorsed by the publisher.
